# Severe SARS‐CoV‐2 infection presenting with acute kidney injury and diabetic ketoacidosis complicated by pancreatitis in a 53‐year man with hypertension

**DOI:** 10.1002/ccr3.3731

**Published:** 2021-01-07

**Authors:** Elrazi Ali, Mohamed Badawi, Ashraf Ahmed, Elabbass Abdelmahmuod, Wanis Ibrahim

**Affiliations:** ^1^ Internal Medicine Department Hamad Medical Corporation Doha Qatar; ^2^ Infectious Disease Department Hamad Medical Corporation Doha Qatar

**Keywords:** acute kidney injury, Diabetic ketoacidosis, pancreatitis, Severe SARS‐CoV‐2

## Abstract

Severe COVID‐19 infection is associated with significant stress and marked immune response that can affect many organs and precipitate DKA, pancreatitis, and acute renal injury, which might be permanent.

## Graphical abstract

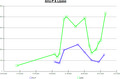

## 1 BACKGROUND

COVID‐19 infection is a newly emerging infectious disease that is not yet fully understood. Usually, it presents with fever, headache, fatigue accompanied by respiratory symptoms like cough and dyspnea, and other systemic involvements. Severe COVID‐19 infection can affect all ages but is seen more with advanced age. Patients with multiple comorbid medical conditions and immunocompromised patients are at risk of developing severe infections. Patients with severe COVID‐19 infection develop acute respiratory distress syndrome (ARDS) and at risk of developing other organ involvement due to deregulated immune response. This could include the kidneys and pancreas heart, liver, and the brain. The occurrence of multiorgan failure results in increased mortality. We present a 53‐year‐old man with a past medical history of hypertension, whose main presentation was severe COVID‐19 infection, found to be in respiratory distress, diabetic ketoacidosis, and acute kidney injury. His hospital course progressed to sepsis, acute pancreatitis, acute kidney injury ending in permanent renal impairment, acute liver failure, and eventually death. Severe COVID‐19 infection is associated with significant stress and marked immune response that can affect many organs and precipitate DKA and acute renal injury, which might be permanent.

Severe acute respiratory syndrome coronavirus 2 (SARS‐CoV‐2) infection was first reported in Wuhan City, China, in December 2019, and it spreads rapidly from one person to another by respiratory droplets.^1^ Daily, we are witnessing different presentations of the disease. Unusual presentations have been reported in association with COVID‐19 infection, symptoms overlapping with malaria infection,^2^ overlapping with HELLP syndrome,^3^ seasonal illnesses like dengue fever,^4^ liver failure,^5^ and precipitating diabetic ketoacidosis (DKA).^6^ DKA is due to insulin deficiency, causing a counter‐regulatory mechanism to break down fatty acids, increasing the production of ketone bodies. DKA can be precipitated by any stressor like myocardial infarction or infections.^7^ The increase in DKA has been linked to recent pandemic of severe acute respiratory distress syndrome (SARS‐CoV‐2). The mechanism remains uncertain; however, it was hypothesized to occur in organs that express SARS cornea virus receptors (angiotensin‐converting enzyme 2 receptors )at the specific organs, like lungs and pancreas, which leads to the cytotoxic effect of these cells and hence its dysfunction.^8^

The understanding of the SARS‐CoV‐2 infection pathogenesis and its effect on the different populations is ongoing. We present a 53‐year man with a past medical history of hypertension presented with severe COVID‐19 infection with diabetic ketoacidosis acute kidney injury (AKI), complicated by the development of liver injury acute pancreatitis, multiorgan failure, and death.

## 2 CASE REPORT

53‐year Bangladeshi male with a past medical history of hypertension presented to the emergency department by ambulance with a history of fever, cough, shortness of breath for 2 days, associated with vomiting once. He had no recent travel or sick contact. There was no clear medication history. On examination, he was conscious, alert, oriented edematous, with basal crackles on chest auscultation. Initial blood investigations showed severe acidosis PH 6.8, ketoacidosis beta‐hydroxybutyrate more than 9.6, and deranged renal parameters (Table 1). Initial serum glucose was normal; repeated glucose measurement showed glucose 16.4 mmol/L. A chest X‐ray showed bilateral pulmonary infiltrates. He required high oxygen by a nonrebreathing mask. He was started on hemodialysis and insulin infusion (DKA protocol) to treat diabetic ketoacidosis. During dialysis, he developed cardiac arrest due to severe bradycardia, was resuscitated, and improved after one cycle of CPR.

**TABLE 1:** The table shows the initial blood investigations on admission

Parameter	Result	Normal range
WBC	28.8 x10^3/uL	4‐10 × 10^3^/μL
ANC	22.3	2‐7 × 10^3^/μL
PLT	341	150‐400 × 10^3^/μL
Hb	13.1 gm/dL	13‐17 gm/dL
Lymph	2.8	1‐3 × 10^3^/μL
eosinophils	0.1	0‐0.5
Urea	23.7	2.8 8.1 mmol/L
Cr	999	62‐106 μmol/L
Glucose	16.4 mmol/L	3.3‐5.5 mmol/L
Na	129	136‐145 mmol/L
K	5.9	3.5‐5.1 mmol/L
Cl	89 mmol/L	89‐107 mmol/L
Hco3	5 mmol/L	22‐29 mmol/L
Ca corrected	2.38 mmol/L	2.15‐2.5 mmol/L
Albumin	33 gm/L	35‐52 gm/L
PH	6.831	7.35‐7.45
PO2	23 mm Hg	83‐108 mm Hg
CO2	38	35‐45 mm Hg
B‐hydroxybutyrate	>9.60 mmol/L	0.03‐0.3 mmol/L
Lactate	15.4 mmol/L	0.5 ‐ 2.2 mmol/L
HbA1c	6.9	Diabetes: 6.5% or Higher
ANCA	Negative	
ANA	Negative	
Anti GBM Ab	Negative	
C3	0.70 gm/L	0.9‐1.8 gm/L
C4	0.30 gm/L	0.1‐0.4 gm/L
Interleukin 6	282 pg/mL	Reference range ≤ 7 pg/mL
Interleukin 2 receptors	23.61 ng/mL	1.2‐ 8.8 ng/mL
Anti‐Mitochondrial Ab	Negative	
Anti‐Mitochondrial M2 Ab	Negative	
Anti‐smooth muscle Ab	Negative	
Urine protein/ Creatinine ratio	147.91 mg/mmol	<=22.60 is high
Ethanol	Less than 2.2	High critical > 44.9
PTH	31 pg/mL	15‐65 pg/mL
Ferritin	191.0 ug/L	30 ‐ 553 ug/L
Triaceylglycerides	1.4 mmol/L	Normal: < 1.7 mmol/L
Fibrinogen	4.32 gm/L	1.7 ‐ 4.20 gm/L
AST	40 U/L	0 ‐ 40 U/L
ALT	20 U/L	0 ‐ 41U/L
procalcitonin	9.43	< 0.5 ng/mL low risk of severe sepsis and/or septic shock > 2.0 ng/mL high risk of severe sepsis and/or septic shock

Then, his saturation worsened, and he was in type 1 respiratory failure. He was on mechanical ventilation on morphine, midazolam, and atracurium. He was in sepsis; urine output was 0.5 mL/kg/h nonoliguric acute kidney injury (AKI). He tested positive for COVID‐19 by the fully automated reverse‐transcription polymerase chain reaction (RT‐PCR) cobas® 6800 (Roche) from nasopharyngeal and throat swabs. Inflammatory markers were elevated and were started on meropenem, vancomycin and received treatment as per local Hamad medical corporation of severe COVID‐19 disease on Tocilizumab, azithromycin 500 mg daily for 7 days, hydroxychloroquine 400 twice daily for the first day then 400 daily for 10 days, Methylprednisolone 40 mg twice daily for 5 days. H score on admission is 51 (AST, fibrinogen).

Further workup, Ultrasound abdomen showed both kidneys with normal size and normal parenchymal echotexture (right kidney measure 11.6 × 4.7 cm and left kidney measure 11.5 × 5.3 cm). There was no calculus or hydronephrosis. The liver was normal in size, measuring 16.2 cm with a mild increase parenchymal echotexture, likely fatty infiltration. The gallbladder is well distended with normal wall thickness. No pericholecystic fluid. No probe tenderness during the examination. Computed Tomography (CT) of the head showed Normal computed tomographic examination of the brain. No evidence of intracranial or subarachnoid hemorrhage or evidence of acute brain parenchymal insult.

He had a prolonged hospital course and was tracheostomized. His renal function did not recover and needed regular hemodialysis. He continued nasogastric tube feeding insulin for blood sugar control. His COVID‐19 was positive throughout the hospital course; only the last result showed COVID‐19 PCR Cycle threshold value 32 (above 30 indicating less infectivity) after 29 days from admission.

The hospital course was complicated by ventilator‐associated pneumonia, sepsis, acute liver injury, and pancreatitis.He was on mechanical ventilation and oxygen requirement was increased from initially 30% to 100%, FIO2 100. There was a rise in bilirubin and raised liver enzymes from initially normal liver enzymes to AST on admission 40 (0‐40 U/L) to more than 7000 and ALT on admission 20 (0‐41 U/L) to 1276 and pancreatic enzymes which were low on admission and shoot later at the end of the disease (Figure 2). WBC from 28.8 × 10^3^/μL on admission to 41 × 10^3^/μL. He received broad‐spectrum antibiotics, and meropenem started on Anidulafungin Ciprofloxacin, Vancomycin tigecycline. Eventually, he passed away due to sepsis and multiorgan failure after 37 days of admission.

## 3 DISCUSSION

**FIGURE 1:**
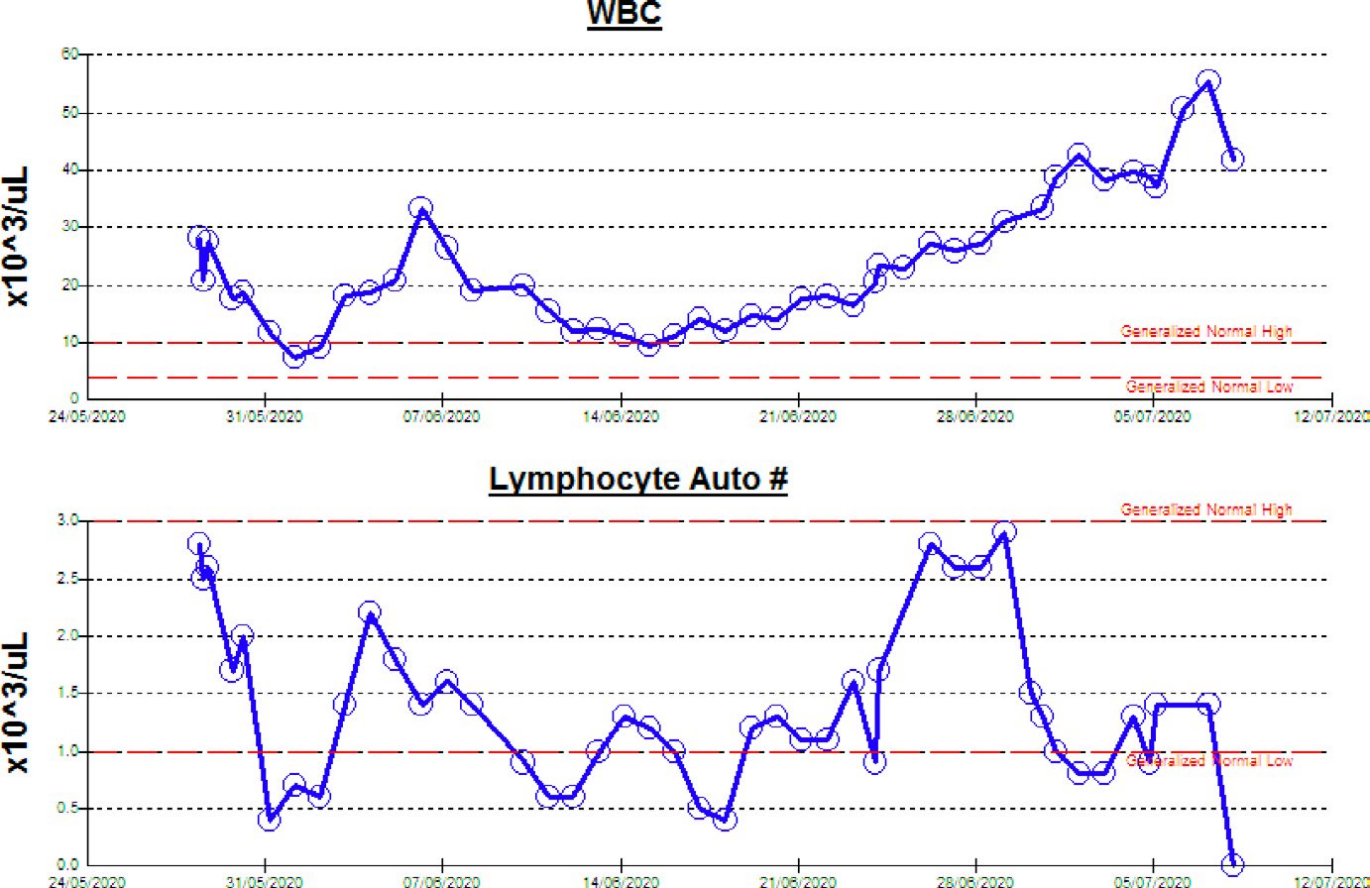
Shows the trend of WBC and lymphocyte count, with rising WBC count and drop of lymphocyte count at the end of the disease

Acute kidney injury is an abrupt decline in kidney function that results in retention of urea and other nitrogenous waste products. In clinical practice, Kidney Disease Improving Global Outcomes (KDIGO) defines AKI as an increase in serum creatinine by ≥ 0.3 mg/dL (≥26.5 micromol/L) within 48 hours or an increase in serum creatinine to ≥ 1.5 times baseline over seven days.^9^ AKI is seen in around 5% of COVID‐19 patients and is associated with high mortality.^10^ Also, as noted in the same study, there was high WBC and low lymphocyte count in patients with kidney injury, as in our patient (Figure 1), with more rise in the WBC count and drop in lymphocytes count to the end of the disease.^10^ Our patient had many factors for renal impairment; he had hypertension and undiagnosed diabetes millets, both are risk factors for the severity of COVID‐19 infection.^11^, ^12^ However, the finding of normal kidneys on abdominal ultrasound and the normal hemoglobin, calcium, and parathyroid hormone on admission supports an acute event.

The mechanism of renal injury in COVID‐19 infection is multifactorial. Firstly, patients may have an underlying renal disease making them vulnerable to AKI. Additionally, in DKA, patients have a loss of intravascular volume, which raises the possibility of prerenal injury, despite being edematous due to loss of the effective intravascular volume. Secondly, prolonged prerenal injury can progress to acute tubular necrosis. Immune‐mediated glomerulonephritis might be there, but biopsy from kidneys of COVID‐19 patients did not reveal such a finding.^13^ Also, the kidney may be injured by the direct cytotoxic effect of the SARS‐CoV‐2 virus, supported by the fact that the virus can enter the cell by angiotensin‐converting enzyme 2,^14^ which is found in the kidneys and further supported by the fact that the virus was detected in the urine of COVID‐19 patients.^15^ It's through these receptors that the virus had resulted in both kidney injury and pancreatic injury resulting in the precipitation of AKI and DKA in this patient. Another explanation for the combined renal and pancreatic injury is the cytokine storm from dysregulated immune response resulted in multiorgan injury including the kidney, liver, and the pancreas.

**FIGURE 2:**
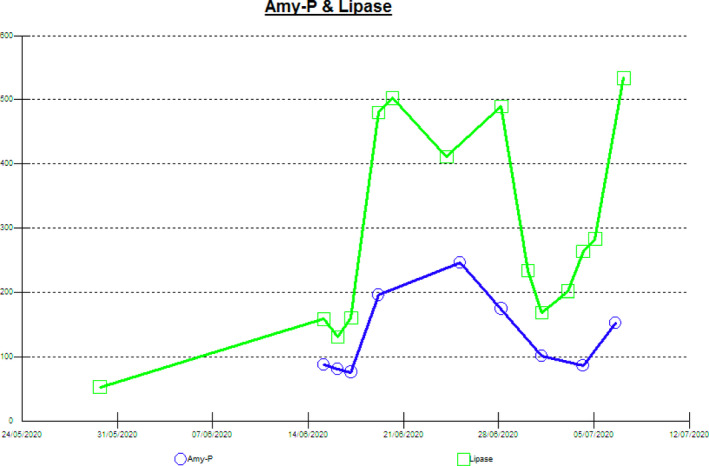
The figure shows the raised pancreatic enzymes complication the hospital course after an initial normal level on admission

The patient had an HbA1c of 6.9 which means he was diabetic but not diagnosed. COVID‐19 infection is reported to be associated with beta‐cell damage and patients present with DKA. Interestingly, the pancreatic injury in COVID‐19 infection seems to happen unrelated to the severity of lung injury as DKA was seen in COVID‐19 infection even without significant respiratory symptoms.^16^ Moreover, DKA frequency and severity are increased during the COVID‐19 pandemic, as reported in Germany.^17^ All these information suggest that diabetic patient are more vulnerable to significant loss of the endocrine function of the pancreas during COVID‐19 infection. Such damage to the Beta cells might be related to several factors. Though the main mechanism of diabetes type one is autoimmunity, it is not known if COVID‐19 can precipitate DKA by inducing an immune response resulting in autoimmune destruction of the pancreas. However, molecular mimicry which plays a pivotal role in the pathogenesis of diabetes type 1 after viral infections might have a comparable role for the SARS‐CoV‐2 virus as for other viruses like the Coxsackie virus.^18^ Up to 7% of patients with COVID‐19 patients had radiological features of pancreatitis on Computed tomography of the abdomen.^19^ Acute pancreatitis in this patient is multifactorial (Figure 2), a possible cause is medications like antibiotics and steroids. The patient was persistently positive for the COVID‐19 until the end of the disease which raises the possibility of COVID‐19 related pancreatitis, similarly due to the high expression of the ACE receptor in the pancreas.^19^

To conclude, COVID‐19 infection can result in any organ involvement, particularly organs with high expression of ACE receptors. Diabetic patients are at risk of significant pancreatic injury, resulting in DKA presentation, absolute insulin deficiency, and pancreatitis. Other organs like kidneys are affected, and damage can be permanent.

## CONFLICT OF INTEREST

All authors have no conflict of interest.

## AUTHOR CONTRIBUTIONS

Elrazi Ali, Mohamed Badawi, Ashraf Ahmed, Elabbass Abdelmahmuod, and Wanis Ibrahim: involved in writing editing final approval.

## ETHICAL CONSIDERATION

The case was approved by the Medical Research Center with MRC‐04‐20‐864.

## Data Availability

Data are available on request.
